# Large Band Gap Narrowing and Prolonged Carrier Lifetime of (C_4_H_9_NH_3_)_2_PbI_4_ under High Pressure

**DOI:** 10.1002/advs.201900240

**Published:** 2019-06-11

**Authors:** Ye Yuan, Xiao‐Fei Liu, Xuedan Ma, Xiaoli Wang, Xin Li, Juan Xiao, Xiaodong Li, Hao‐Li Zhang, Lin Wang

**Affiliations:** ^1^ Center for High Pressure Science and Technology Advanced Research Shanghai 201203 China; ^2^ State Key Laboratory of Applied Organic Chemistry College of Chemistry and Chemical Engineering Lanzhou University Lanzhou 730000 China; ^3^ Center for Nanoscale Materials Argonne National Laboratory 9700 South Cass Avenue Lemont IL 60439 USA; ^4^ School of Physics and Electronic Engineering Linyi University Linyi 276005 P. R. China; ^5^ Department of Physics Fudan University Shanghai 200433 China; ^6^ Institute of High Energy Physics Chinese Academy of Sciences Beijing 100049 China

**Keywords:** 2D organic–inorganic halide perovskites, band gap narrowing, carrier lifetime prolongation, high pressure, phase transitions

## Abstract

Due to their superior optical and electronic properties and good stability, 2D organic–inorganic halide perovskites (OIHPs) exhibit strong potential for optoelectronic applications. However, the large band gap, short carrier lifetime, and high resistance hinder their practical performance. In this work, the band gap is successfully tuned, the carrier lifetime is prolonged, and the resistance of (C_4_H_9_NH_3_)_2_PbI_4_ (BA_2_PbI_4_) is reduced directly using high pressure. The band gap is decreased to less than 1 eV at 35.0 GPa, and the highest pressure is studied. The carrier lifetime at 9.9 GPa is 20 times longer than that at ambient conditions. Moreover, the resistance is reduced by four orders of magnitude at 34.0 GPa accompanying band gap narrowing. This work indicates that pressure plays an effective role in tuning the optical and electronic structures of BA_2_PbI_4_, and also provides a strategy to synthesize high‐performance OIHP materials.

Recent years have witnessed the strong resurgence of interest in 3D organic–inorganic halide perovskites (OIHPs), with the general formula ABX_3_, sparked by their great potential applications in solar cells.^1^, ^2^, ^3^, ^4^ The power‐conversion efficiency of 3D OIHPs has increased to over 20%, which is close to other commercial rivals, like polycrystalline silicon and cadmium telluride (CdTe). The excellent photovoltaic performance of 3D OIHPs is contributed by their efficient light absorption, modest charge‐carrier mobility, and long carrier lifetime‐prompted long diffusion length.^5^, ^6^, ^7^ However, the low thermal stability^8^, ^9^ and low resistance to moisture^10^, ^11^ of 3D OIHPs have hampered their commercialization. Therefore, other kinds of new organic–inorganic halide perovskites with superior properties are highly desirable for further applications.

Unlike 3D OIHPs, 2D OIHPs often keep the general formula of ABX_4_ (organic monolayer) or A′_2_BX_4_ (organic bilayer), where A is a divalent organic cation, A′ is a monovalent organic cation, B is a metal cation, and X is a halide anion.^12^ The hydrophobic molecules in the organic layers effectively improve the moisture resistance compared to the 3D perovskite, which solves the main obstacle of 3D OIHPs.^13^, ^14^ Organic and inorganic layers stack alternatively in the crystal, which can be treated as a quantum‐well‐like structure.^15^, ^16^ The alternating layers of 2D lead‐halide perovskites induce an exceptionally large exciton binding energy^15^, ^17^ and a wide band gap in contrast to 3D lead‐halide perovskites, which limits its optical performance. The much shorter carrier lifetime of 2D lead‐halide perovskites also impedes its practical application. Therefore, reducing the band gap and prolonging the carrier lifetime are two critical issues to be considered when improving the performance of 2D lead‐halide perovskites for practical applications.

Hydrostatic pressure can dramatically affect the structure and tune the electronic properties of materials,^18^, ^19^, ^20^ which have been proven in many 3D OIHPs systems.^21^, ^22^, ^23^, ^24^, ^25^ For example, Wang et al. studied the structural transition and photoresponse of CH_3_NH_3_PbBr_3_ (MAPbBr_3_) under pressure.^21^ Their results suggest that hydrostatic pressure can significantly affect the crystal structure and photovoltaic related properties of MAPbBr_3_. Wang et al. observed that pressure can efficiently reduce the band gap of formamidinium lead bromide (FAPbBr_3_)^22^ and methylammonium lead chloride (MAPbCl_3_).^23^ Lv et al. found that the structural stability and photoresponsiveness of CH_3_NH_3_SnI_3_ enhanced after pressure‐induced amorphization and recrystallization.^24^ Therefore, by combining the structural stability of 2D perovskites and pressure effects, better optoelectronic performance is expected at high pressure.^26^, ^27^, ^28^, ^29^ BA_2_PbI_4_ is an important 2D OIHP, and its structure and optical properties transition have been attracting a lot of attention.^30^, ^31^, ^32^ Matsuishi et al.^31^ and Liu et al.^30^ studied the band gap transition of BA_2_PbI_4_ and observed several transitions during compression. However, these new phases were not well characterized, and their relationship to the properties is especially unclear. Very recently, Yin et al.^32^ carried out experiments and theoretical calculations to study the structural and optical properties of BA_2_PbI_4_ exfoliated nm‐thin flakes at pressure <10 GPa and found two phase transitions at around 0.1 and 1.4 GPa. Yin et al. also studied the photoluminescence (PL) lifetime of BA_2_PbI_4_ nm thin flakes. After the first phase transition, the PL lifetime increased but after the second phase transition, the PL lifetime decreased. Until now, the optical and electrical performance transition of bulk BA_2_PbI_4_ at higher pressure has not been reported. Herein, we studied the optical and electrical behavior of BA_2_PbI_4_ under high pressure using in situ UV–vis absorption, PL, time‐resolved PL, and impedance measurements. It is clear that pressure can significantly narrow the band gap, prolong carrier lifetime, and reduce the resistance of BA_2_PbI_4_. Our studies suggest a potential application of BA_2_PbI_4_ at high pressure and also inspire a new strategy to obtain better performance organic–inorganic halide perovskite material.

UV–vis absorption measurements were carried out on BA_2_PbI_4_ from ambient conditions to around 40 GPa to study the high‐pressure effects on the band gap. The absorption spectra are shown in **Figure** **1**a. The measured band gaps with the corresponding microphotographs of the sample at different pressures are shown in Figure 1b. At ambient conditions, BA_2_PbI_4_ is translucent orange, and the band gap is about 2.28 eV (Figure 1d), which is much bigger than the ideal band gap (1.34 eV) in Shockley theory.^33^ During compression, three dramatic changes of the band gap were observed from the high‐pressure absorption spectra (Figure 1a). At near 0.22 GPa, the band gap dramatically jumps to about 2.37 eV (Figure 1c,e). The sample color correspondingly changes to translucent yellow from orange accompanying the transition, as seen in Figure 1b. At 2.2 GPa, a new absorption edge was observed at about 2.06 eV from the spectrum, as marked by the arrow in Figure 1a, indicating a second phase transition happened in the sample, and the new phase has a smaller band gap. From the optical image shown in Figure 1b, it is clear that part of the sample turned red at 2.2 GPa. After the sudden initial reduction, the band gap continued narrowing with the increasing pressure (Figure 1c). At 13.1 GPa, a third phase transition occurred, and a new edge was observed from the absorption spectrum. The band gap continued decreasing with pressure up to 40 GPa. At near 27.5 GPa, the band gap reduced to 1.25 eV, which is close to the ideal band gap in Shockley theory.^33^ Hence, at around 27.5 GPa, BA_2_PbI_4_ may exhibit excellent photovoltaic performance. The measurements were carried out up to about 40 GPa, and the minimum band gap we could accurately observe was about 0.95 eV at around 35 GPa. The band gap narrowed with a surprising percentage of 58.3% compared to that at ambient conditions.

**Figure 1:**
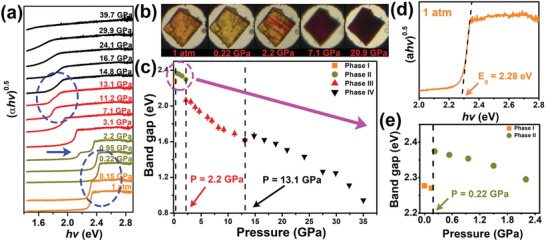
a) In situ high‐pressure absorption spectra of BA_2_PbI_4_. b) Optical images of BA_2_PbI_4_ at selected pressures. c) Variations of the BA_2_PbI_4_ band gap as a function of pressure. d) The absorption spectrum of BA_2_PbI_4_ at ambient conditions. e) Variations of the of BA_2_PbI_4_ band gap below 2.2 GPa.

Previous studies on the electronic structure of 2D and 3D perovskite materials have suggested that the organic cation has no direct effect on the electronic properties, and the band gap of perovskite mainly depends on BX_6_ octahedra instead.^12^, ^34^, ^35^ The valence band maximum (VBM) corresponds to the antibonding hybrid state formed between the B‐s and X‐p orbitals, and the conduction band minimum (CBM) is determined by a nonbonding hybrid state between the B‐p and X‐p orbitals.^34^, ^36^ Therefore, tuning the BX_6_ octahedra structure can significantly change the band gap of perovskite materials. The B–X bond length shortening enhances B‐s and X‐p orbital coupling, which can push up the VBM and narrow the band gap. However, the B–X bond angle decreases (i.e., octahedral tilting), which can reduce the B‐s and X‐p orbital coupling and widen the band gap.^25^ Therefore, we can deduce that the pressure‐induced Pb–I bond length shortening makes the band gap gradually narrow, whereas the dramatic band gap jump at 0.22 and 2.2 GPa is from the PbI_6_ octahedra structural transition. As the band gap increased after the first phase transition, the PbI_6_ octahedra of phase II tilted more, while the sharp band gap reduction after the second phase transition indicated that the PbI_6_ octahedra of phase III became less tilted. At ambient pressure, BA_2_PbI_4_ crystallizes in the orthorhombic phase with space group *Pbca*.^37^ It transforms to *Pbca* at 274 K,^37^ and the distortion of the PbI_6_ octahedra at low temperature enlarges the band gap of BA_2_PbI_4_, which also makes the color of BA_2_PbI_4_ turn yellow. Considering the tiny intrigued pressure of the first phase transition during the compression and band gap transition, we deduced that phase II corresponds to the low‐temperature phase at ambient pressure.

We also studied the in situ high‐pressure photoluminescence and time‐resolved PL to trace the optical properties and carrier recombination of BA_2_PbI_4_. From the PL measurements, both the emergence of new peaks (**Figure** **2**a) and peak shift versus pressure (Figure 2b) agreed well with the results observed in the high‐pressure absorption measurements. As shown in Figure 2a, the PL peak centered at 525.5 nm at ambient conditions. After the first transition, the enlarged band gap made the PL peak sharply shift to 505.1 nm. As the pressure reached 2.6 GPa, the newly emerged peak centered at 575.9 nm corresponded well to the new absorption band gap. However, we also observed the PL peak from phase II (Figure 2a), which indicated that it was a mixed phase. This phase II PL peak decreased dramatically after 2.6 GPa and eventually vanished at 7.1 GPa (Figure 2c). At 9.2 GPa, we also observed a new weak PL peak (marked by the red star in Figure 2a), which further proves the third phase transition observed in the high‐pressure absorption measurements.

**Figure 2:**
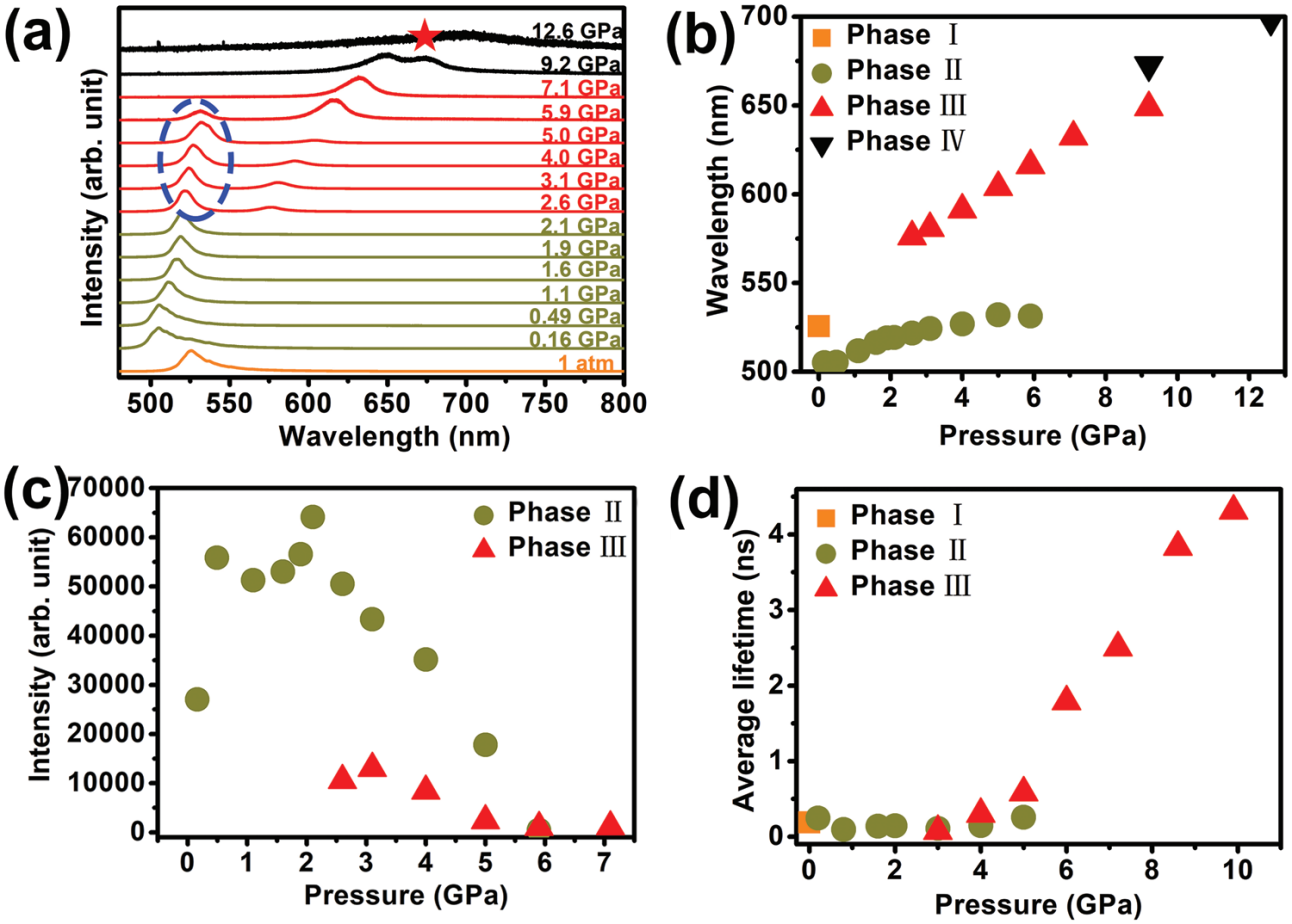
a) In situ high‐pressure photoluminescence spectra of BA_2_PbI_4_. b) Variations of the PL peak center of BA_2_PbI_4_ as a function of pressure. c) Variations of the PL peak intensity of BA_2_PbI_4_ as a function of pressure. d) Variations of the carrier lifetime of BA_2_PbI_4_ as a function of pressure.

The exciton lifetime of BA_2_PbI_4_ as a function of pressure is shown in Figure 2d. The exciton lifetime of BA_2_PbI_4_ at ambient conditions, 0.8 and 9.9 GPa are shown in Figure S2 of the Supporting Information. The measured PL decay curves were fitted using triple exponential functions. Due to the short lifetimes, the instrument response function was reconvoluted during the fitting process. At ambient conditions, the mean exciton lifetime is 189 ps, which is slightly longer than the Yin et al. reported result (≈150 ps). As their sample is an exfoliated flake, the greater structural defective states in their sample might lead to a shorter lifetime. After the first phase transition, accompanying the band gap enlarging, the carrier lifetime reduced to 95 ps (0.8 GPa). The larger band gap deepens trap states; therefore, a significant drop in the lifetime can be observed (Figure 2d). However, after the second phase transition, Yin et al. observed a longer lifetime (190 ps) at 0.4 GPa. This difference might be related to crystal size and morphology. Previous studies have proven that electron–hole pair recombination can be efficiently suppressed by the dominant trap states, located in the shallow energy near CBM and VBM.^38^, ^39^ Pressure‐induced band gap narrowing makes the trap states even shallower. Thus, a larger portion of recombination becomes radiative, and a longer carrier lifetime is consequently expected. From 0.8 to 5.0 GPa, the carrier lifetime of phase II increased from 95 to 257 ps. Phase III of BA_2_PbI_4_ is more sensitive to high pressure and its lifetime is easily tuned. After the second transition, the carrier lifetime almost linearly increased from ≈0.07 ns at 3.0 GPa to ≈4.31 ns at 9.9 GPa. For higher pressure, the PL was too weak to be detected.

To study the mechanism of the optical variation of BA_2_PbI_4_ under high pressure, we carried out XRD measurements on BA_2_PbI_4_ during compression up to 25.5 GPa. The ambient structure of BA_2_PbI_4_ was confirmed via Rietveld refinement of the XRD pattern, with details shown in **Figure** **3**b. At 0.17 GPa, several new rings appeared and the first reflection from 2.57° dramatically shifted to 2.67° (Figure 3a,c). Based on the color change and tiny intrigued pressure of the first phase transition, we deduced that this was an isostructural transition and the new phase corresponds to the low‐temperature *Pbca* phase at ambient pressure. We used the low‐temperature phase structure to refine the structure, which agrees with the observed spectra (Figure 3c). At ambient conditions, BA_2_PbI_4_ displays a K_2_NiF_4_ structure type. The [PbI_6_] group formed a corrugated inorganic layer. Two layers of interdigitated butylammonium cations are embedded between the inorganic sheets. The organic and inorganic layers interact via the N—H···I hydrogen bond in the *c* direction. After the first phase transition, the [PbI_6_] group rotated along the *a* direction, making the inorganic layer more staggered. The organic cations kept their initial formation but rearranged after the phase transition. The rearrangement of the organic cation induced the decrease of interlayer spacing, which made the (002) diffraction ring shift (Figure 3d).

**Figure 3:**
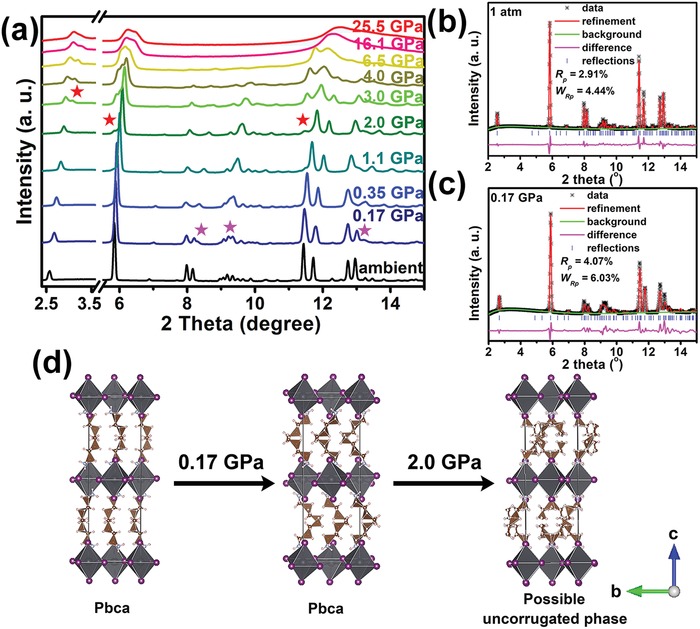
In situ XRD measurements of BA_2_PbI_4_ under high pressures. a) Integrated XRD pattern of BA_2_PbI_4_ during the compression and decompression cycle. b,c) Detailed variations of the first and second transition. d) Schematic crystal structures of BA_2_PbI_4_ before and after the first phase transition.

At about 2.0 GPa, three new reflections appeared at ≈3.01°, 5.84°, and 11.52° (Figure 3a), respectively, which suggested a second phase transition occurred. These new peak intensities also enhanced with increasing pressure. However, we could also still observe the diffraction rings from phase II. This result proves that only part of the sample changed after 2.0 GPa. Similar to BA_2_PbI_4_, (C_2_H_5_NH_3_)_2_CuCl_4_ is also a typical type of A_2_BX_4_ crystal with the same structure of *Pbca* at ambient conditions. At near 4.2 GPa, the (020) Bragg peak of (C_2_H_5_NH_3_)_2_CuCl_4_ starts to split into two peaks, which indicates that it transforms to *P2_1_/a*, a subgroup of *Pbca*.^40^ Besides this material, some other layered organic lead iodide perovskites that have longer organic chains ((C*_n_*H_2_ *_n_*_+1_NH_3_)PbI_4_, *n* = 5–10), all transform to *P2_1_/a* from *Pbca* with the enhancement of inorganic distortion at a lower temperature.^37^, ^41^ Therefore, BA_2_PbI_4_ transforming to *P2_1_/a* at ≈2 GPa seems to be reasonable. However, transforming to a *P2_1_/a* phase indicates that the tilt degree of PbI_6_ octahedra enhanced, which leads to a wide band gap.^37^ This conflicts with our interpretation of the absorption measurements. Therefore, our results favor that bulk BA_2_PbI_4_ became a less tilted phase near 2.0 GPa, whereas nm‐thick BA_2_PbI_4_ becomes a *P2_1_/a* phase,^32^ which is probably due to the crystal size and morphology effect.

Unlike BA_2_PbI_4_, (C_6_H_13_NH_3_)PbI_4_ transforms from the *Pbca* phase (phase 3) to another orthorhombic phase (phase 2) and a new tetragonal phase (phase 1) at 354.9 and 371.3 K, respectively.^37^ The chain‐melting transition was presumed to be the mechanism of a phase III to phase II transition. The phase transition from II to I was attributed to a conformational disorder of the organic chains. After the transition, gauche‐type kinks run through the organic chains. Similar order‐to‐disorder transitions have also been observed in other compounds, such as (C_8_H_17_NH_3_)PbI_4_ and (C_10_H_21_NH_3_)PbI_4_.^41^ After the transition, the inorganic layers become uncorrugated. The samples' band gaps reduce and the color changes to red accompanying the transition. For BA_2_PbI_4_, the observed second phase transition might correspond to the *Pbca* phase‐to‐uncorrugated phase transition. As shown in Figure 3d, there are only weak van der Waals forces between the two adjacent cations. Therefore, the organic layer easily becomes disordered under high pressure. Pressure might push these two organic sheets to insert into each other, and the chains might kink and twist together. This ordered‐to‐disordered transition could induce the sudden reduction of interlayer spacing, and uncorrugated inorganic layers (Figure 3d), which could well explain the newly emerged small *d*‐spacing (002) diffraction peak and sharply decreased band gap after the transition.

With the increasing pressure, the sharp diffraction rings became weak and broad, and several weak peaks disappeared after 6.5 GPa, which could be induced by the disordered degree of organic layers enhancement. At higher pressure, and especially after the third phase transition, only five broad peaks were left. The broader and weaker peak of the (002) diffraction ring also suggests that the third phase corresponds to the amorphization of BA_2_PbI_4_. Very recently, Li et al. reported an optical study of Cs_2_AgBiBr_6_ under high pressure.^42^ They found that the band gap of Cs_2_AgBiBr_6_ started to decrease above 6.5 GPa. By combining experimental and theoretical results, they speculate that the structural amorphization contributed to the band gap narrowing. Similar to the result of Li et al., the structural amorphization of BA_2_PbI_4_ after the third phase transition also contributed to the continuous band gap narrowing under high pressure.

We further performed theoretical simulations to study the electrical structures of phase I and phase II of BA_2_PbI_4_. The calculated band structures (Figure S3a, Supporting Information) and projected density of states (PDOS) (Figure S3b, Supporting Information) show a complete same trend as the experiment. The calculated PDOS indicates that the states near the Fermi energy level are mainly contributed by I and Pb atoms. The calculation also indicates that the BX_6_ octahedra distortion enhancement could reduce the B‐s and X‐p orbital coupling, which further support the band gap changes discussed above.

To further explore the electrical transport behavior of BA_2_PbI_4_ under high pressure, high‐pressure impedance spectra were measured. The two‐electrode method was used for measuring the impedance spectra. Detailed information is illustrated in the Supporting Information. As shown in Figure S1a of the Supporting Information, at ambient conditions, a semicircle occurred at a higher frequency and an inclined upward straight tail at lower frequency could be observed, which suggested BA_2_PbI_4_ presents ionic transport behaviors.^43^, ^44^, ^45^, ^46^ Early works on OIHPs have also provided evidences for ionic conduction, in agreement with our observation on BA_2_PbI_4_.^47^ Notably, at 3.1 GPa, the inclined upward straight lines tend to bend towards the *Z* ^′^ axis (Figure S1b, Supporting Information), which indicated that pressure induced the ionic transport behavior to electrical transport behavior transition. As discussed, the XRD experiment indicated the BA_2_PbI_4_ started to transform into an uncorrugated phase at about 2.0 GPa, became dominant at around 3 GPa, and completed at ≈6 GPa. Thus, the observed ionic transport to electrical transport behavior transition should be induced by the phase transition when phase III becomes dominant. As shown in **Figure** **4**b, the resistance of BA_2_PbI_4_ increased with increasing pressure from ambient pressure to 6 GPa. Above 6 GPa, the resistance started to decrease following the onset of the disorder degree enhancement of the organic layers (Figure 4b). After the third phase transition, structural amorphization was led by PbI_6_ octahedral distortion. Meanwhile, the resistance sharply reduced with the increasing pressure (Figure 4a,b). Jaffe et al.^48^ and Li et al.^42^ reported that similar structural amorphization contributed to the continuous band gap narrowing at higher pressure before. Jaffe et al. deduced that the increases in the Pb–I bond compression, and structural changes on a more local scale, play roles in the band gap and conductivity variation. Li et al. tried to simulate the band gap evolution under compression without considering amorphization. However, the huge conflict between their experimental and calculation results made them speculate that structural amorphization is the underlying mechanism for sustaining band gap narrowing. As our sample is similar to theirs, we agree that structural amorphization contributed to the continuous band gap narrowing and resistance dropping. The resistance quickly decreased accompanying the structural changes on a local scale (Figure 4b). At around 34 GPa, the resistance is almost 10 000 times smaller, which proves that the electrical performance of BA_2_PbI_4_ is better at high pressure.

**Figure 4:**
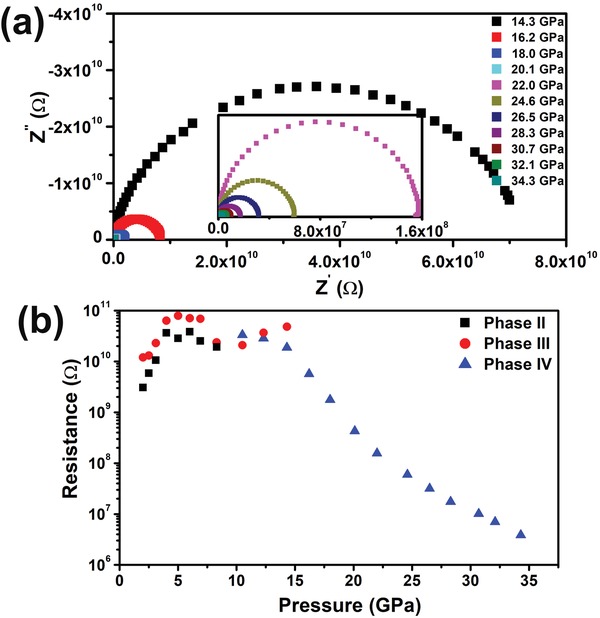
a) Nyquist impedance spectra of BA_2_PbI_4_ at 14.3 to 34.3 GPa. The inset shows the Nyquist impedance spectra of BA_2_PbI_4_ at 22.0 to 34.3 GPa. b) Pressure dependence of the resistance of BA_2_PbI_4_.

Pressure is a proven effective method to improve the performance of organic perovskite materials.^21^, ^22^, ^23^, ^25^, ^48^ However, due to their low structural stability, the enhancement is limited. Like MAPbBr_3_,^21^ and FAPbI_3_,^49^ FAPbBr_3_,^22^ the band gaps widened and the resistance and carrier lifetime increased below 3 GPa, which suggests that the pressure‐induced enhancement is limited. Kong et al. calculated that if MAPbI_3_ could keep the *I4/mcm* phase near 1.5 GPa, the band gap would reduce to 1.3 to 1.4 eV, which achieves the Shockley–Queisser limit and makes the energy‐conversion efficiency of solar cells increase to 33%.^25^ **Table** **1** summarizes the variation of the resistance and carrier lifetimes of several other organic perovskites and BA_2_PbI_4_ at high pressure. Compared to other organic perovskite materials, the pressure effects on the band gap and carrier lifetime of BA_2_PbI_4_ are tremendous. The band gap decreased over 1.352 eV at high pressure. The carrier lifetime at 9.9 GPa was 20 times longer than that at ambient conditions. The significantly better performances of BA_2_PbI_4_ at high pressure suggest its potential for practical applications. However, the reversibility of the high‐pressure phase hinders its application at ambient conditions. Our studies show the structural information and physical properties of BA_2_PbI_4_ at high pressure, which could guide people to synthesize the high‐pressure phase at ambient conditions for further application.

**Table 1:** Comparison of the pressure‐induced changes in the band gap and carrier lifetimes of several halide perovskites

	*E*_0_	*E*_min_	Δ	Δ/*E*_0_ [%]	*T*_0_	*T*_max_	Δ/*T*_0_ [%]	Ref.
MAPbI_3_	2.258	0	2.258	100	112.38	225.24	100.04	25, 48
MAPbBr_3_	2.361	2.32	0.041	1.7	41.09	45.07	9.7	21
MAPbCl_3_	3.06	3.01	0.05	1.6				23
FAPbI_3_	1.489	1.337	0.152	10.2	12.61	14.9	18.2	49
FAPbBr_3_	2.27	2.1	0.17	7.5				22
BA_2_PbI_4_	2.302	<0.95	>1.352	>58.7	0.189	4.312	2181.5	This study

In summary, we studied the optical behavior and structural evolution of BA_2_PbI_4_ under high pressure. Three phase transitions were observed with pressure up to around 40 GPa. The band gap decreased from 2.28 eV at ambient conditions to 0.95 eV at near 35 GPa, accompanying the evolution, which is a narrowing of over 58%. Pressure can also effectively prolong the carrier lifetime. At 9.9 GPa, the carrier lifetime is 20 times longer than that at ambient conditions. At 34.3 GPa, the resistance is almost 10 000 times smaller. The improved performance of BA_2_PbI_4_ suggests its potential applications under high pressure, and also offers a mode to synthesize new OIHPs with better performance under high pressure.

## Conflict of Interest

The authors declare no conflict of interest.

## Supporting information

Supplementary
