# Supplementary material for: Large Band Gap Narrowing and Prolonged Carrier Lifetime of (C4H9NH3)2PbI4 under High Pressure
Source: Adv Sci (Weinh). 2019 Jun 11;6(15):1900240. doi: 10.1002/advs.201900240 (PMC6685472; doi:10.1002/advs.201900240)
Supplement: Supplementary file 1 — Supplementary [file ADVS-6-1900240-s001.pdf]

Copyright WILEY-VCH Verlag GmbH & Co. KGaA, 69469 Weinheim, Germany, 2019.

ADVANCED SCIENCE Open Access

Supporting Information

for *Adv. Sci.*, DOI: 10.1002/advs.201900240

Large Band Gap Narrowing and Prolonged Carrier Lifetime of $(C_4H_9NH_3)_2PbI_4$ under High Pressure

Ye Yuan, Xiao-Fei Liu, Xuedan Ma, Xiaoli Wang, Xin Li, Juan Xiao, Xiaodong Li, Hao-Li Zhang, and Lin Wang**

Supplemental Material

Large band-gap narrowing and prolonged carrier lifetime of $(C_4H_9NH_3)_2PbI_4$ under high pressure

Ye Yuan[1*], Xiaofei Liu[2*], Xuedan Ma[3*], Xiaoli Wang,[4] Xin Li[1,5], Juan Xiao[2], Xiaodong Li[6], Haoli Zhang[2#] and Lin Wang[1#]

[1] Center for High Pressure Science and Technology Advanced Research, Shanghai 201203, China

[2] State Key Laboratory of Applied Organic Chemistry, College of Chemistry and Chemical Engineering, Lanzhou University, Lanzhou 730000, China

[3] Center for Nanoscale Materials, Argonne National Laboratory, 9700 South Cass Avenue, Lemont, Illinois 60439, United States

[4] Institute of Condensed Matter Physics, Linyi University, Linyi 276005, People's Republic of China

[5] Department of Physics, Fudan University, Shanghai 200433, China

[6] Institute of High Energy Physics, Chinese Academy of Sciences, Beijing 100049, China

[*] Y. Y., X. L. and X. M. contribute equally to this work

[#] To whom correspondence may be addressed. Email: haoli.zhang@lzu.edu.cn wanglin@hpstar.ac.cn

High pressure impedance measurement

Impedance spectra were measured *via* using a Solartron1260 impedance analyzer and 1296 dielectric interface. The powder sample was loaded in a Mao-type symmetric DAC with a pair of 300-μm culets and placed in cBN gasket hole with a diameter on the order of 150 μm. 2 μm thick Pt foil was used as electrodes. A two-electrode configuration was used for measuring the impedance spectra. Two electrodes were attached to the top and bottom culet, respectively. Cubic boron nitride powder mixed with epoxy was used for the insulation between the platinum electrode and metal gasket. Impedance spectra were collected from 1×10^{-2} Hz to 1×10^{6} Hz.

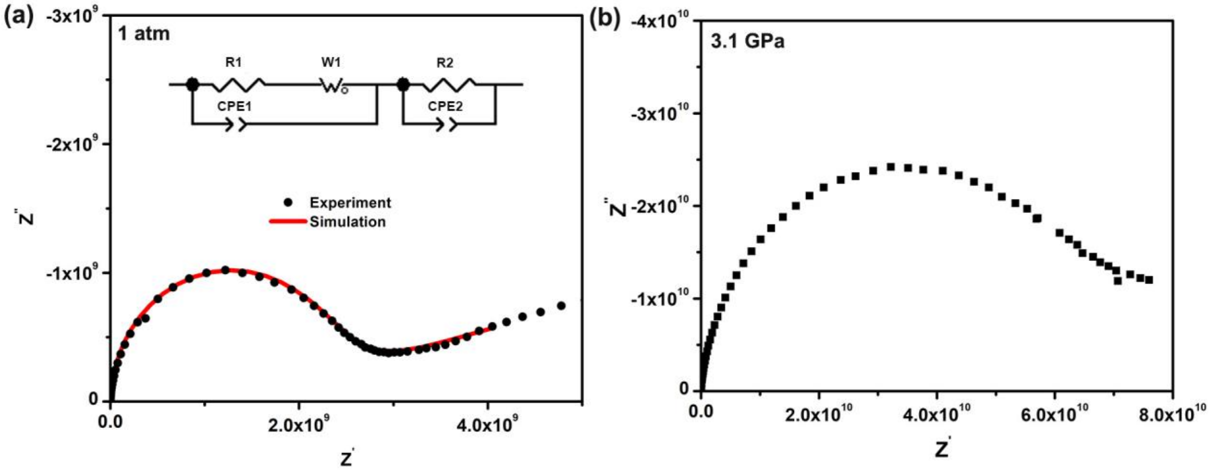

Figure. S1. Impedance data of BA_2PbI_4 at ambient conditions (a) and 3.1 GPa (b).

High pressure absorption measurement

In situ high-pressure UV-vis absorption spectroscopy measurements were performed on an UV-vis absorption spectrophotometer with a response time of 1 s. Absorption spectra (250−1000 nm) were measured using a deuterium−halogen light source. The sample was loaded in a Mao-type symmetric DAC with a pair of 300-μm culets and placed in T301 steel gasket hole with a diameter on the order of 150 μm. Silicon oil was used as pressure-transmitting medium. The silicone oil and diamond signal was recorded as background.

High pressure PL measurement

PL spectra were collected by using a Horiba LabRAM HR Evolution Raman spectrometer with a 473 nm laser as the excitation. The sample was loaded in a Mao-type symmetric DAC with a pair of 400-μm culets and placed in T301 steel gasket hole with a diameter on the order of 200 μm. Silicon oil was used as pressure-transmitting medium.

High pressure PL lifetime measurement

In situ high-pressure time-resolved photoluminescence measurement was conducted at the Center for Nanoscale Materials (CNM), ANL. 400 nm laser was used as excitation. The sample was loaded in a Mao-type symmetric DAC with a pair of 400-μm culets and placed in T301 steel gasket hole with a diameter on the order of 200 μm. Silicon oil was used as pressure-transmitting medium. The measured PL decay curves were fitted using triple exponential functions. Due to the short lifetimes, the instrument

response function (IRF) was reconvoluted during the fitting process. The mean PL time was calculated *via* $<\tau> = (A_1*\tau_1^2+A_2*\tau_2^2+A_3*\tau_3^2)/(A_1*\tau_1+A_2*\tau_2+A_3*\tau_3)$.

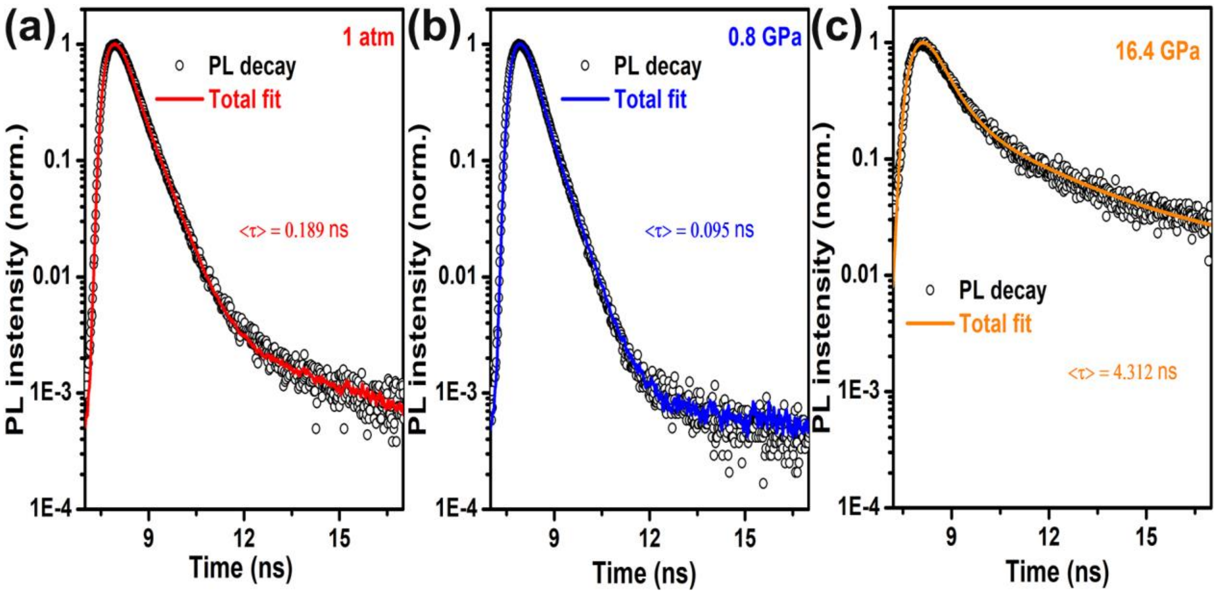

Figure. S2. Carrier lifetime of BA_2PbI_4 at ambient conditions (a), 0.8 GPa (b), and 16.4 GPa (c)

In Situ Synchrotron High Pressure Powder XRD

In situ synchrotron high pressure powder XRD experiments were carried out at 4W2 beamline of Beijing Synchrotron Radiation Facility (BSRF). Monochromatic X-ray with wavelength of 0.6199 Å was employed. The powder sample was loaded in a Mao-type symmetric DAC with a pair of 300-μm culets and placed in T301 steel gasket hole with a diameter on the order of 150 μm.

GSAS Refinement

We firstly used LeBail mode to make sure the simulated peak position well match with the that of obseved peak. Then, we used Rietveld mode to better refine the data.

Theoretical simulation

The underlying *ab initio* structural relaxations and electronic properties calculations are performed in the frame work of density functional theory within generalized gradient approximation Perdew-Burke-Ernzerhof (GGA-PBE), as implemented in the VASP code. The projector augmented wave (PAW) pseudopotentials are adopted with the PAW potentials taken from the VASP library. The cutoff energy

(600 eV) for the expansion of the wave function into plane waves and Monkhorst-Pack *k*-meshes (*k*-points density 0.02 Å^{-1}) are chosen to ensure that all the energy calculations are well converged to better than 1 meV/atom.

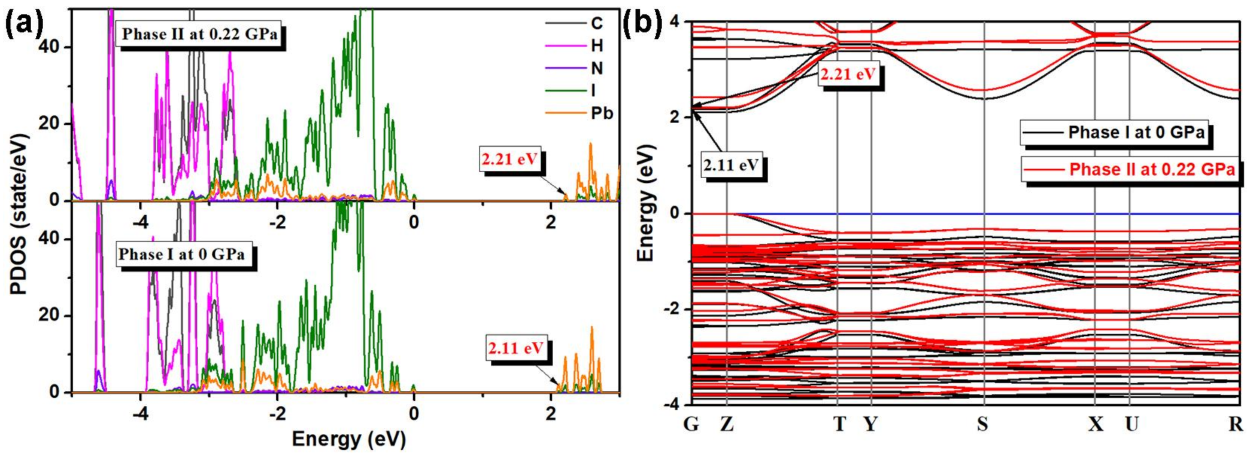

Figure S3. (a) Calculated projected density of states. (b) Calculated band structures of phase I and phase II at 0 GPa 0.22 GPa, respectively.
